# New Insights on the “DC Shock-Reperfusion” in ST Elevation Myocardial Infarction: Killing Two Birds with One Stone?

**DOI:** 10.4274/balkanmedj.2016.0447

**Published:** 2017-08-04

**Authors:** Ümran Özdamar, Mehmet Kadri Akboğa, Muhammed Fatih Bayraktar, Özcan Özeke

**Affiliations:** 1 Clinic of Cardiology, Türkiye Yüksek İhtisas Training and Research Hospital, Ankara, Turkey

## To The Editor,

Coronary artery plaque rupture and subsequently thrombus formation are the most common cause of interruption of coronary flow in patients with ST-segment elevation myocardial infarction (STEMI). According to the current guidelines of STEMI management, coronary flow has to be achieved as soon as possible to prevent ischaemic complications. Ischaemia is one of the main trigger mechanisms of arrhythmias, including primary ventricular fibrillation in patients with STEMI. Primary ventricular fibrillation may occur within 5-10 minutes after acute coronary total occlusion. Although mortality rates decrease dramatically with effective defibrillation via direct current shock, one-third of STEMI patients die because of primary ventricular fibrillation within the first 2 hours after the onset of symptoms. Primary ventricular fibrillation is less often seen after reperfusion. Even though the certain treatment is defibrillation, undesirable effects of electroshock, such as myocardial damage, can occur. The mechanism of reperfusion and primary ventricular fibrillation is not known from all aspects. Regardless of the mode of treatment, Pezza (1) suggested that there could be a relation between direct current shock and reperfusion in STEMI. 

**Figure 1:**
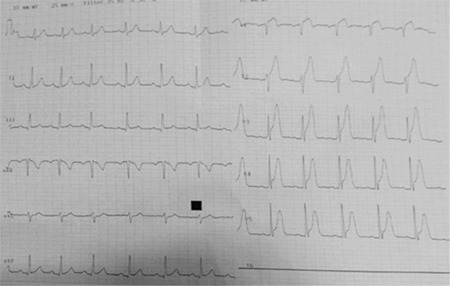
Lead electrocardiogram showing ST segment elevation in precordial leads.

A 45-year-old man who complained of chest pain lasting as long as 30 minutes with ST segment elevation in anterior derivation of conventional 12-lead electrocardiography (ECG) was presented to our emergency department (Figure 1). The patient was taken to the angiography laboratory for primary percutaneous coronary intervention as soon as possible after heparin, acetylsalicylic acid and ticagrelor had been administered. Shortly after we had seen the thrombotic image revealing total occlusion in the left anterior descending artery during angiography, primary ventricular fibrillation occurred (video 1, video 2). The patient was immediately defibrillated with 200 joules direct current shock and sinus rhythm was provided. In the next scene of angiography there was no thrombotic image and spontaneous coronary flow was seen in left anterior descending artery (video 3). After successful electrical defibrillation, the patient’s symptoms, including chest pain, regressed and repeated ECG showed prominent ST-segment resolution. Recently, Yayla et al. (2) demonstrated ST-segment resolution on ECG after defibrillation for primary ventricular fibrillation in three STEMI cases. In our case we demonstrated dissolution of coronary artery thrombus after defibrillation for primary ventricular fibrillation by angiography prior to primary percutaneous coronary intervention in a patient with anterior myocardial infarction to whom antiplatelet and anticoagulant therapy were administered. The higher concentrations of endothelial-derived micro particles in primary ventricular fibrillation due to acute coronary occlusion compared with STEMI without rhythmic disturbances suggests different patterns of acute coronary occlusion (3). The high-energy direct current shock can lead to fragmentation of the thrombus that is completely blocking the coronary artery and facilitate its clearance via the endogenous fibrinolytic system. There are some case reporting in literature reporting that primary ventricular fibrillation patients with marked precordial ST-segment elevation showed ST-segment normalization during the hospital stay with features suggestive of Brugada pattern or other channelopathies (4). Therefore, ST-segment elevation without a dynamic and evolving acute myocardial infarction underscores the need to consider other causes of ST-segment elevation. However, some ST elevation and primary ventricular fibrillation combination reports have been explained by coronary spasm or only direct current shock-induced ST elevation (5), but the possibility of our hypothesis deserves consideration in these cases. We can speculate that urgent direct current shock may not only be an effective treatment of fatal arrhythmic primary ventricular fibrillation but might also have a role in the re-establishment of spontaneous circulation by the dissolution of the coronary thrombus. Written informed consent has been obtained from the patient.

The videos related to the manuscript can be accessed from our website.
